# Supplementary material for: Isoform-specific knockdown of long and intermediate prolactin receptors interferes with evolution of B-cell neoplasms
Source: Commun Biol. 2023 Mar 20;6:295. doi: 10.1038/s42003-023-04667-8 (PMC10027679; doi:10.1038/s42003-023-04667-8)
Supplement: Supplementary file 2 — Supplementary Information [file 42003_2023_4667_MOESM2_ESM.pdf]

Supplementary Materials (Taghi Khani *et al.*)

Isoform-specific knockdown of long and intermediate prolactin receptors interferes with evolution of B-cell neoplasms

Inventory

- Supplementary Figures 1-17
- Supplementary Tables 1 and 2

Supplementary Fig. 1: *Gating strategy for flow cytometry analysis of mouse splenic WBCs.*

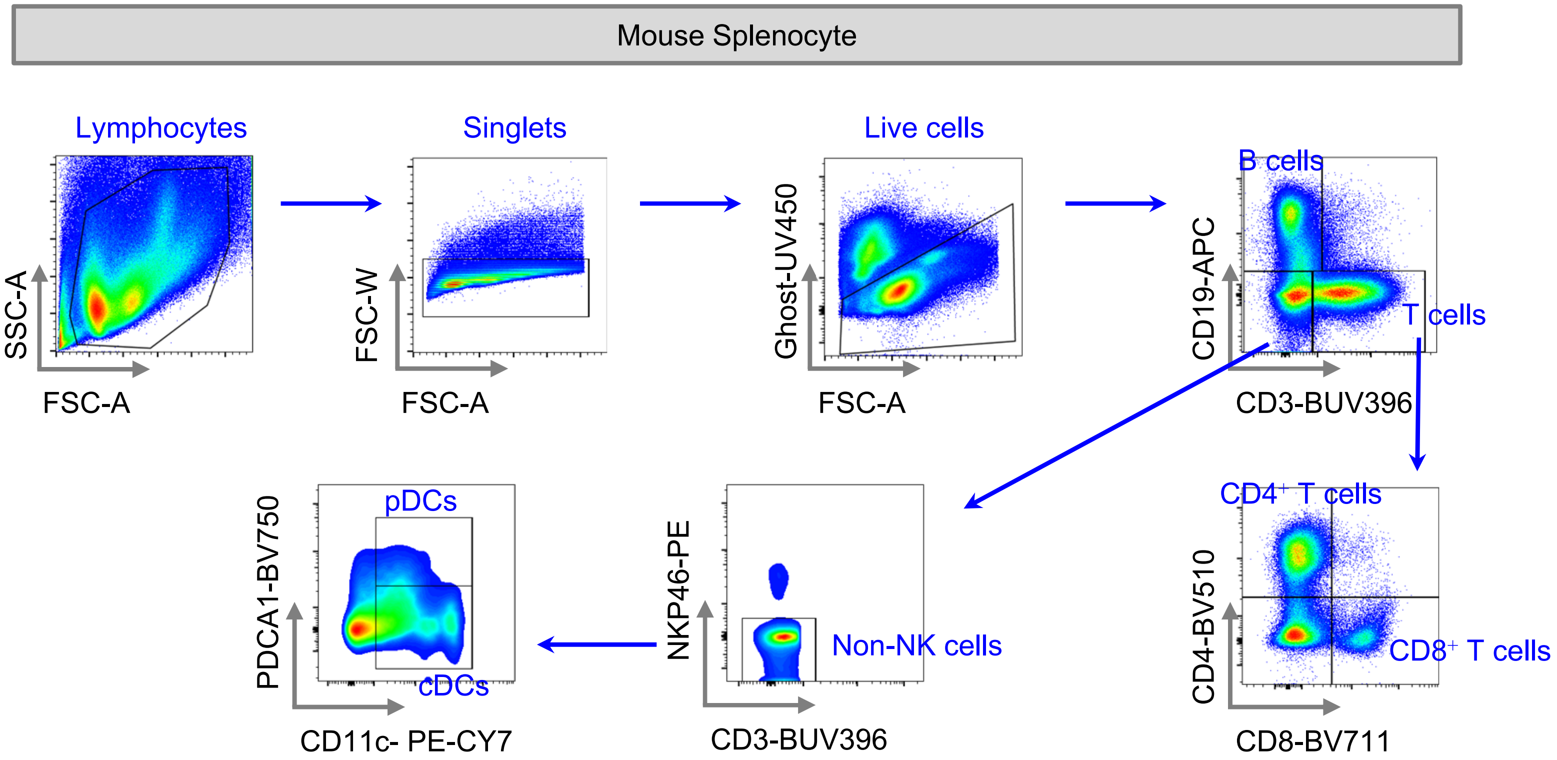

Supplementary Fig. 1: Gating strategy for flow cytometry analysis of mouse splenic WBCs. From the lymphocyte cluster; singlets were gated followed by selection of live (Ghost-UV450^{-}) populations. B cells, T cells, and non-B non-T cells were then gated on CD19^{+}, CD3^{+}, and CD19^{-} CD3^{-}, respectively. T cells were gated on CD8^{+}, CD4^{+}, and CD4^{-}CD8^{-} T cells. pDCs and cDCs were gated on CD11c and PDCA1 after gating out NKp46^{+} NK cells from the non-B non-T fraction.

Supplementary Fig. 2: *LFPRLR knockdown did not induce significant changes in numbers of T cell subsets in SLE prone mice.*

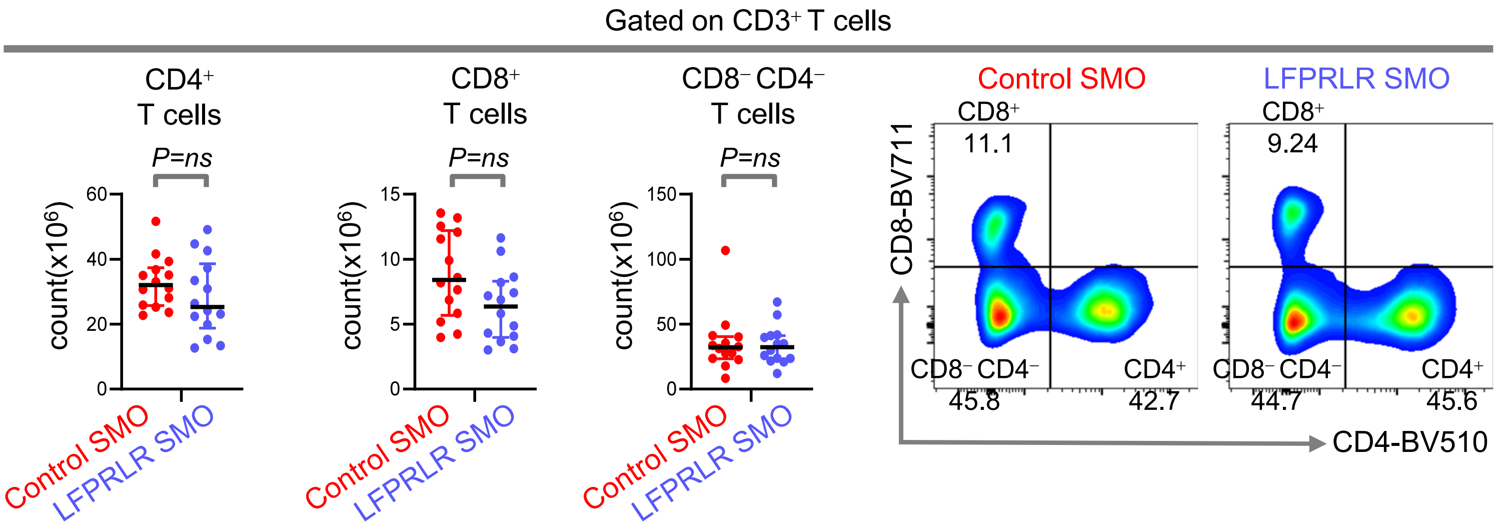

Supplementary Fig. 2: LFPRLR knockdown did not induce significant changes in frequencies or numbers of T cell subsets in SLE-prone mice. Quantitation of numbers and representative flow cytometry plots of $CD4^+$, $CD8^+$, and $CD4^-CD8^-$ T cells of *MRL-lpr* SLE prone mice treated with control SMO (n=14, red) or LFPRLR SMO to knockdown LFPRLR (n=14, blue). Graphs show median ± interquartile range. Exact p-values were calculated using the Mann-Whitney U test. ns = non-significant.

Supplementary Fig. 3: *Gating strategy for flow cytometry by intracellular staining.*

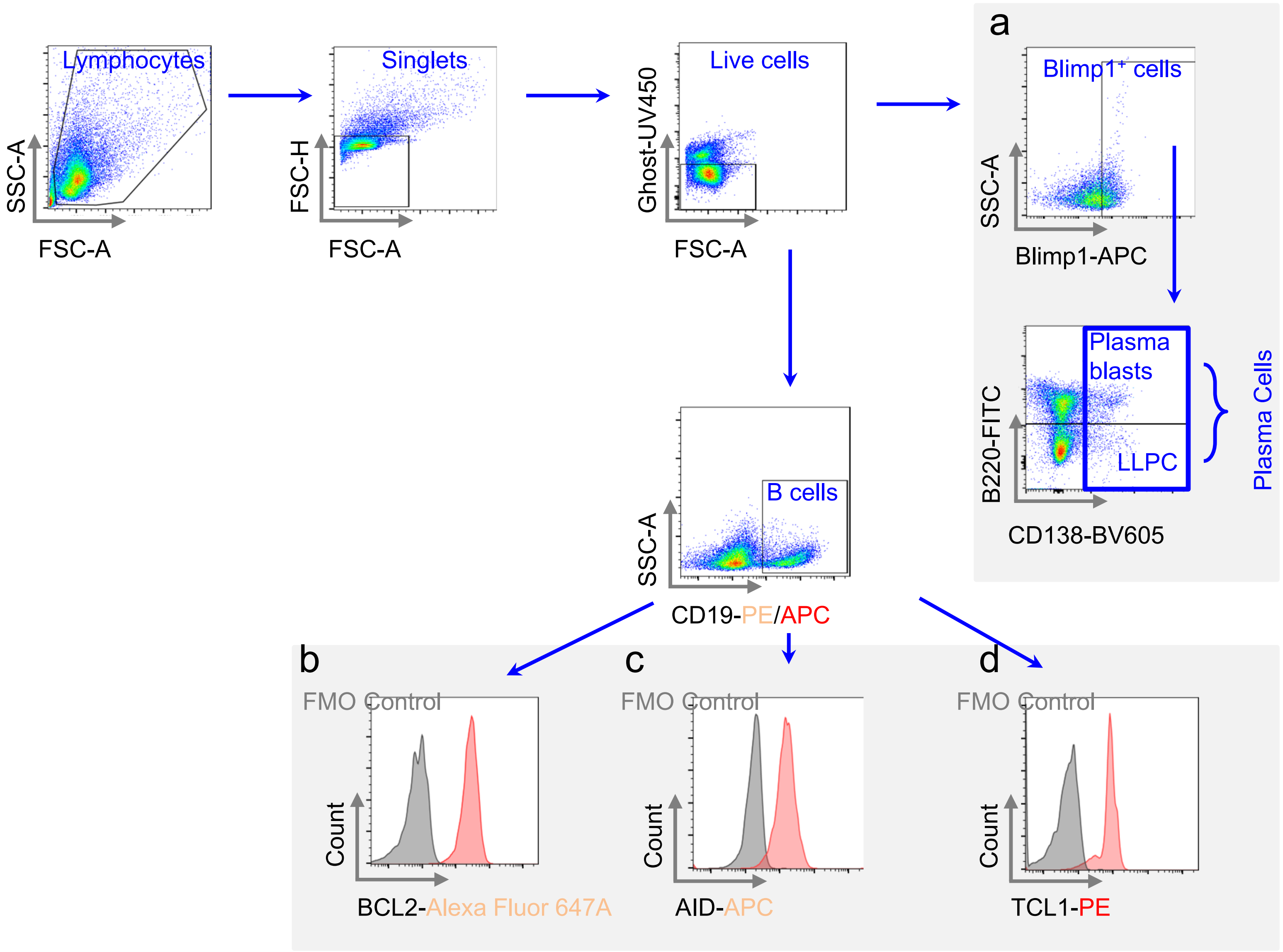

Supplementary Fig. 3: Gating strategy for flow cytometry by intracellular staining. From the lymphocyte cluster; singlets were gated followed by selection of live (Ghost-UV450^{-}) populations. (**a**)Total CD138^{+} plasma cells, CD138^{+}B220^{+} plasmablasts, and CD138^{+}B220^{-} LLPC were then measured by gating on Blimp1^{+}cells. (**b-c**) Total CD19^{+} B cells were then gated and BCL2 **(b)** AID **(c)** or TCL1 **(d)** was measured within these cells. Gates for all markers were set based on fluorescence minus one controls (FMO).

Supplementary Fig. 4: *Anti-dsDNA autoantibody production trended towards a reduction after LFPRLR knockdown*

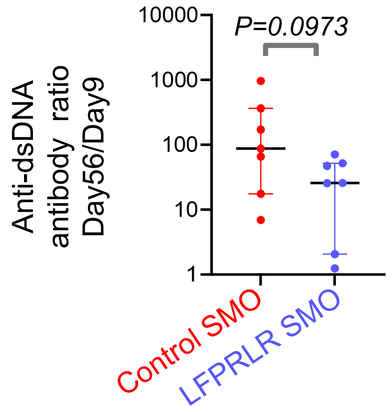

Supplementary Fig. 4: Anti-dsDNA autoantibody production trended towards a reduction after LFPRLR knockdown. Fold change/ increase in anti-dsDNA antibodies (day 56: day 9) in mice treated with either control SMO (n=7) or LFPRLR SMO (n=7). Exact p-values were calculated using the Mann-Whitney U test.

Supplementary Fig. 5: *Gating strategy for flow cytometry analysis and magnetic sorting of mouse splenic B cells*

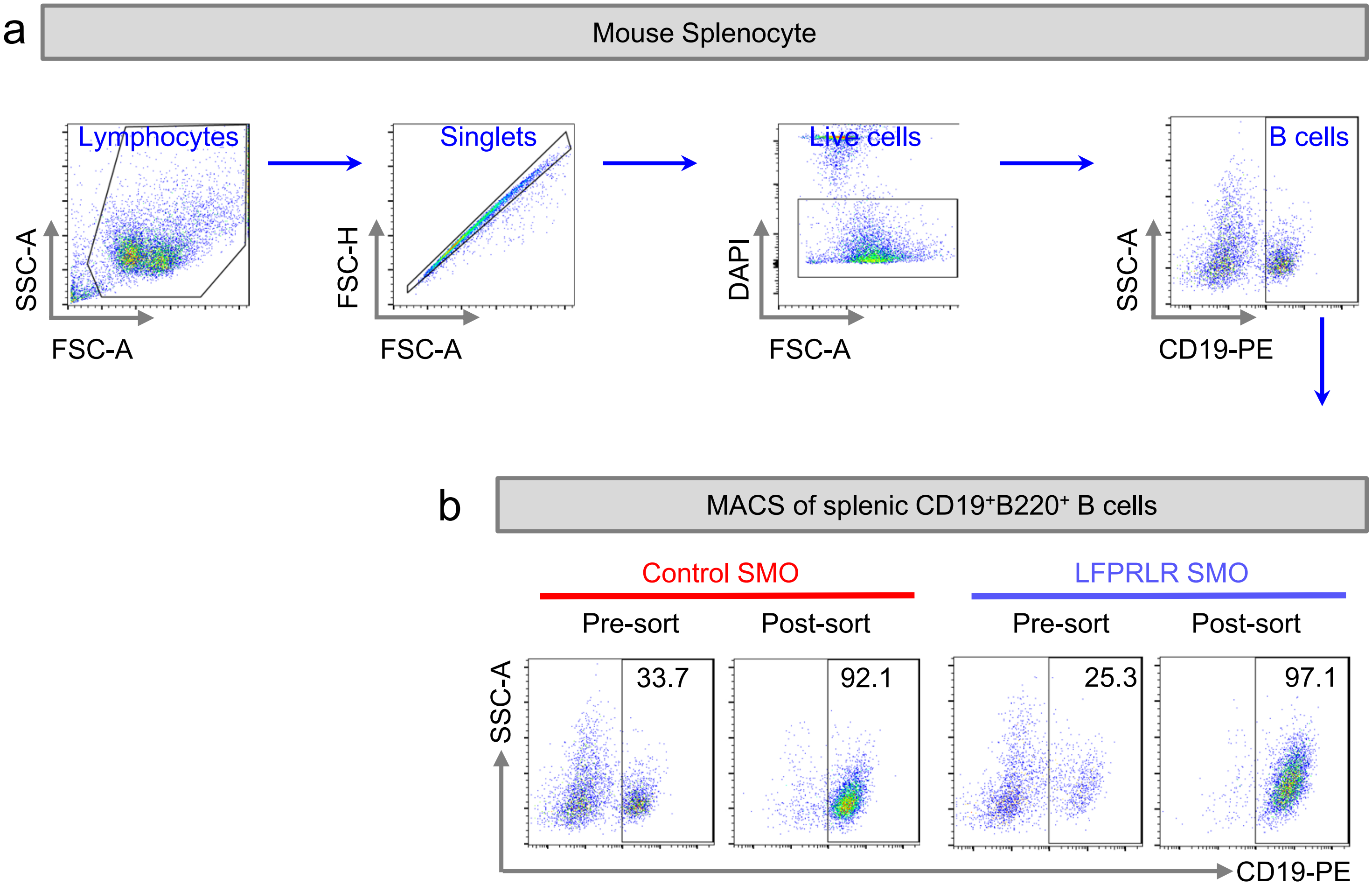

Supplementary Fig. 5: Gating strategy for flow cytometry analysis and magnetic sorting of mouse splenic B cells. (a) From the lymphocyte cluster; singlets were gated followed by selection of live (Ghost-UV450⁻) populations. B cells were then gated on CD19⁺. **(b)** Flow cytometry verification of splenic B cells sorted out magnetically using CD19 microbeads from one representative 14-week-old *MRL-lpr* SLE-prone mouse treated with either control SMO (n=14) or LFPRLR SMO (n=14) for 8 weeks.

Supplementary Fig. 6: *LFPRLR knockdown did not induce significant changes in numbers of T cell and DC subsets in DLBCL prone mice.*

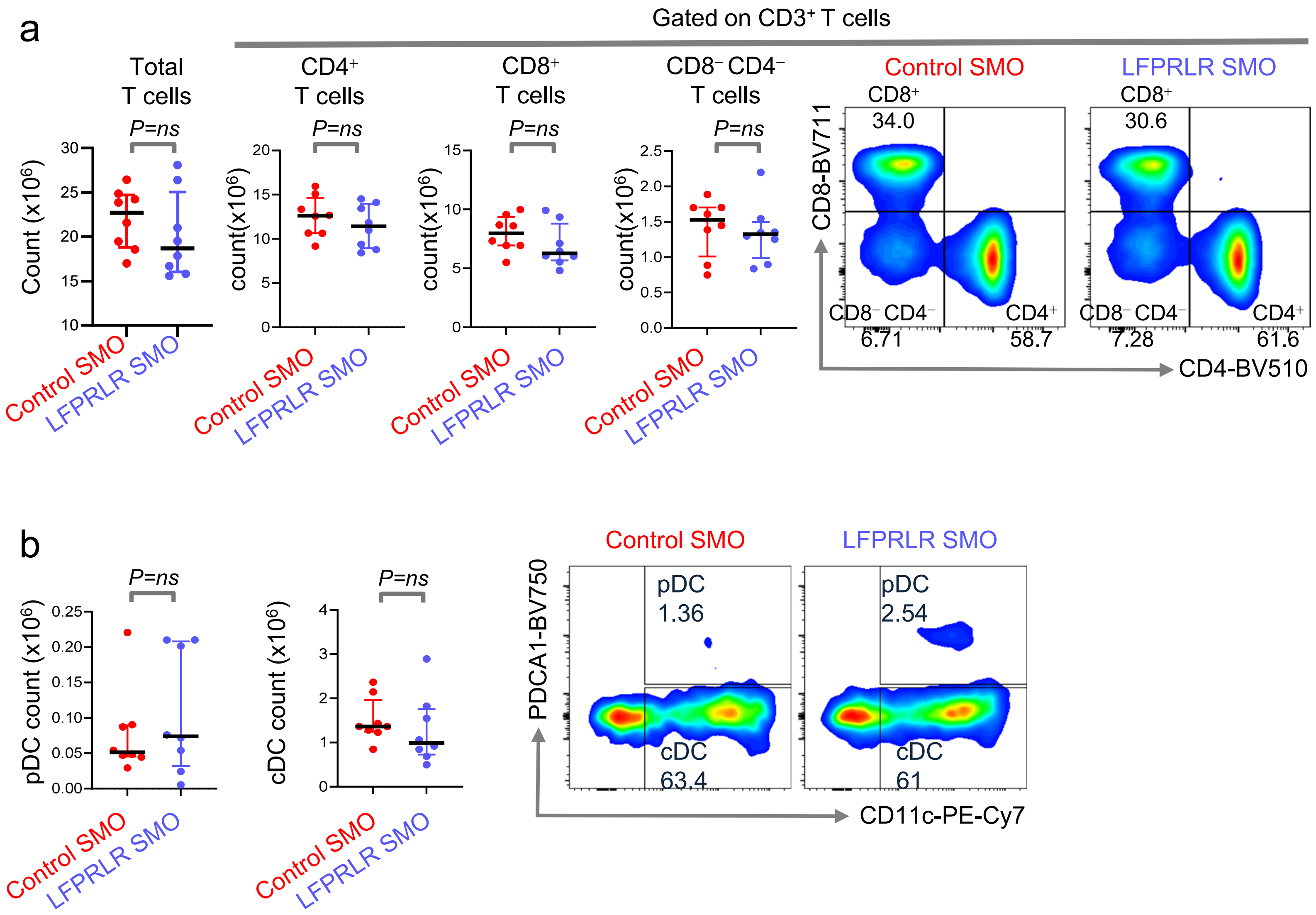

Supplementary Fig. 6: LFPRLR knockdown did not induce significant changes in numbers of T cell and DC subsets in DLBCL-prone mice. (a,b) Quantitation of numbers and representative flow cytometry plots of total, $CD4^{+}$, $CD8^{+}$, and $CD4^{-}CD8^{-}$ T cells (**a**) and $CD11c^{+}PDCA1^{+}$ pDCs and $CD11c^{+}PDCA1^{-}$ cDCs (**b**) in *TCL1*-tg DLBCL-prone mice treated with control SMO (n=8, red) or LFPRLR SMO to knockdown LFPRLR (n=8, blue). Graphs show median ± interquartile range. Exact p-values were calculated using the Mann-Whitney U test. ns = non-significant.

Supplementary Fig. 7: *Knockdown of LFPRLR in DLBCL-prone mice does impact cycling of splenic B cells*

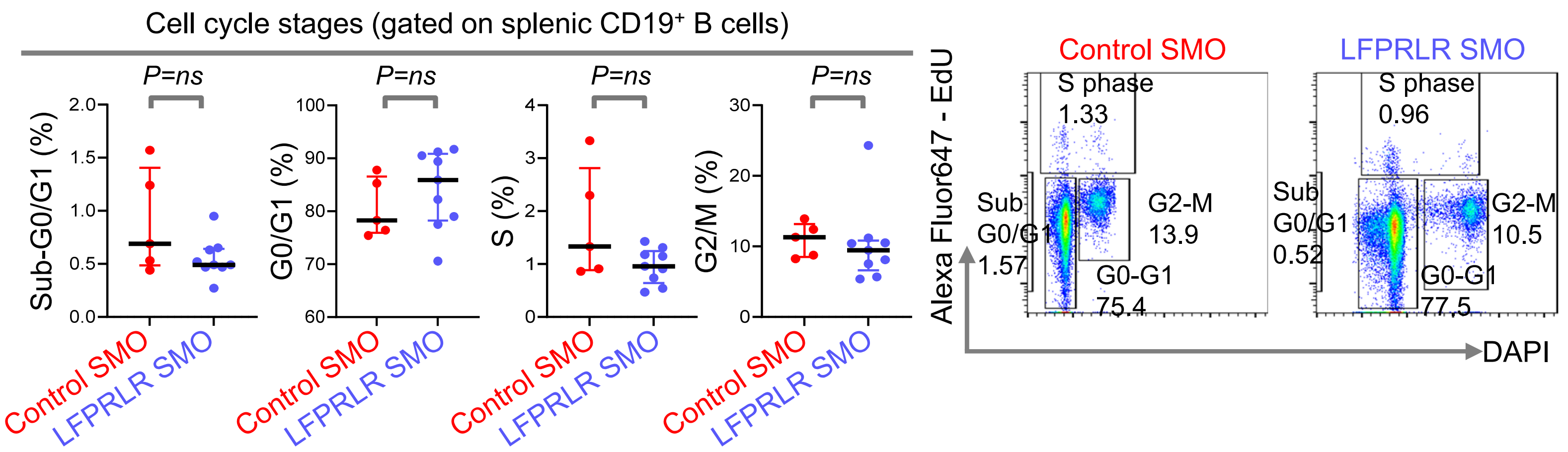

Supplementary Fig. 7: Knockdown of LFPRLR in DLBCL-prone mice does not impact cycling of splenic B cells. Percentages of B cells in sub G0/G1, G0/G1, S, G2/M phases and representative flow cytometry in spleens of *TCL1-tg* mice treated with control SMO (n=8, red) or LFPRLR SMO (n=8, blue). Graphs show median ± interquartile range. Exact p-values were calculated using the Mann-Whitney U test. ns = non-significant.

Supplementary Fig. 8: *Knockdown of LFPRLR reduced Bcl2 expression but does not alter Myc expression in total splenic WBCs of DLBCL-prone mice*

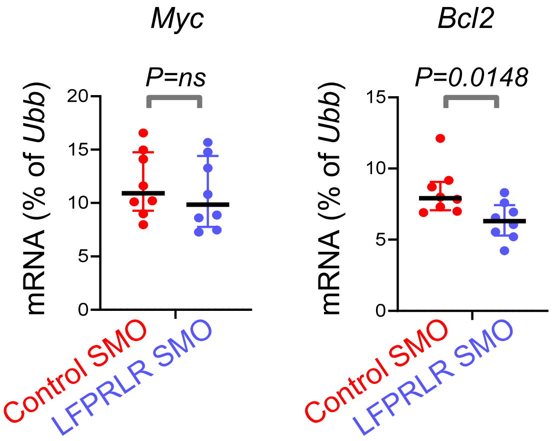

Supplementary Fig. 8: Knockdown of LFPRLR reduced *Bcl2* expression but does not alter *Myc* expression in total splenic WBCs of DLBCL-prone mice. Comparison of total splenocyte transcript levels of *Myc* and *Bcl2* by qPCR in *TCL1-tg* mice treated with control SMO (n=8, red) or LFPRLR SMO (n=8, blue). Graphs show median ± interquartile range. Each dot in qPCR analyses of transcripts corresponds to the mean expression of that gene in one mouse calculated from 3 technical replicates. Exact p-values were calculated using the Mann-Whitney U test. ns = non-significant

Supplementary Fig. 9: *Total PRLR expression in diagnosis and treated DLBCL patients does not predict clinical outcome*

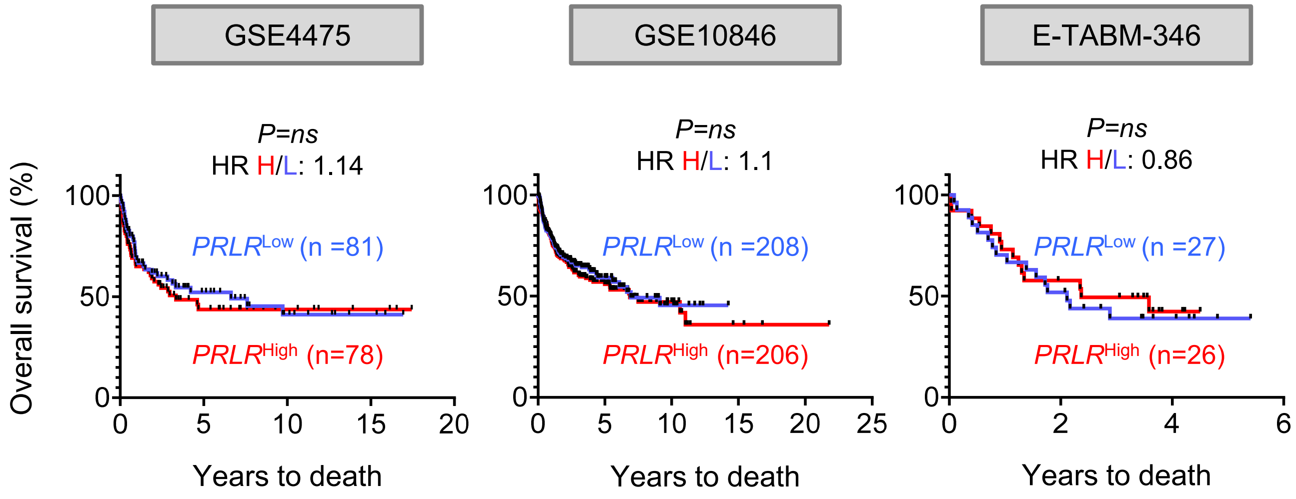

Supplementary Fig. 9: Total *PRLR* expression in diagnosis and treated DLBCL patients does not predict clinical outcome. Comparison of overall survival probabilities of patients with B cell lymphomas from 3 datasets (GSE4475, GSE10846, and E-TABM-346) divided into two groups as *PRLR*^{High} and *PRLR*^{Low} based on the median expression of total *PRLR* mRNA. GSE4475 includes 123 DLBCL and 36 BL patients at diagnosis. GSE10846 includes samples from 414 DLBCL patients collected after treatment with Rituximab-CHOP or CHOP and E-TABM-346 includes samples from 53 DLBCL patients collected after treatment with Rituximab-CHOP or CHOP. P values were measured by Log-rank (Mantel-Cox) test. ns=not significant. HR= Hazard Ratio.

Supplementary Fig. 10: *Normal B cells always express both SFPRLR and LF/IF PRLR but 50% of BCL2/MYC-driven B-ALL express only LF/IF PRLR*

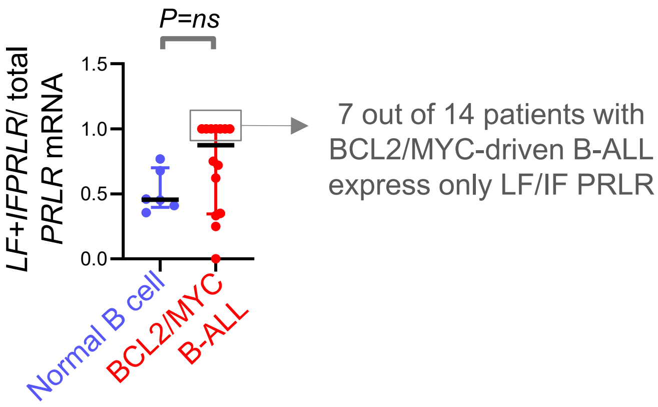

Supplementary Fig. 10: Normal B cells always express both *SFPRLR* and *LF/IF PRLR* but 50% of BCL2/MYC-driven B-ALL express only *LF/IF PRLR*. Comparison of expression of ratio of *LF+IF*: total *PRLR* mRNA between normal B cells and patients with BCL2/MYC-driven B-ALL from the St. Jude RNA sequencing cohort EGAS00001003266. Graph shows median ± interquartile range. Exact p-value was calculated using the Mann-Whitney U test. ns = non-significant.

Supplementary Fig. 11: *Gating strategy for flow cytometry analysis and magnetic sorting of human peripheral blood B ,T and NK cells*

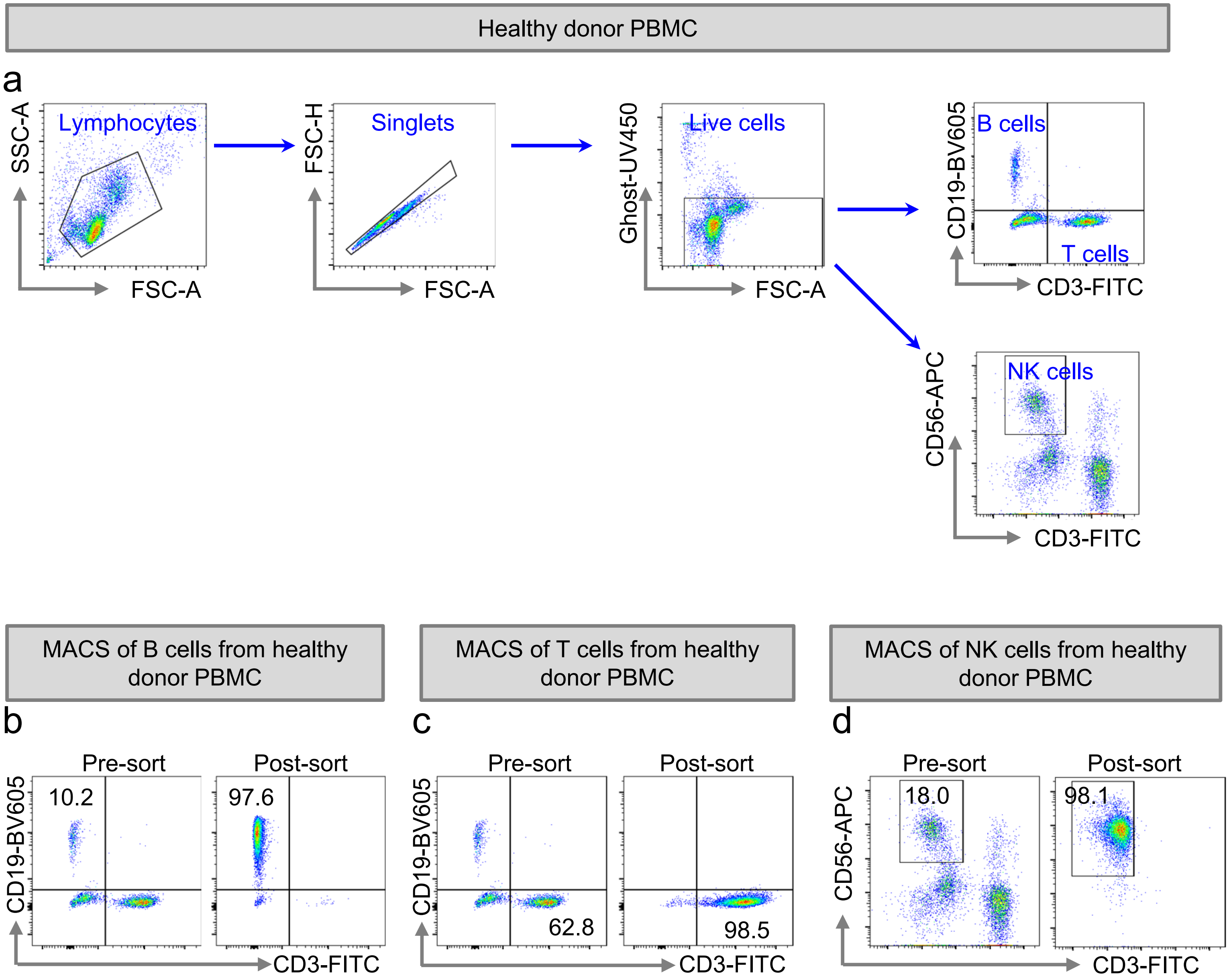

Supplementary Fig. 11: Gating strategy for flow cytometry analysis and magnetic sorting of human peripheral blood B, T and NK cells. (a) From the lymphocyte cluster; singlets were gated followed by selection of live (Ghost-UV450⁻) populations. B cells were then gated on $CD3^{-}CD19^{+}$, T cells were gated on $CD3^{+}CD19^{-}$ and NK cells were then gated on $CD3^{-}CD56^{+}$. **(b-d)** Verification of post-sort purity of magnetically sorted B (b), T (c) and NK (d) cells from PBMC of healthy donors by flow cytometry.

Supplementary Fig. 12: *Knockdown of LFPRLR in malignant human B cells reduces their viability*

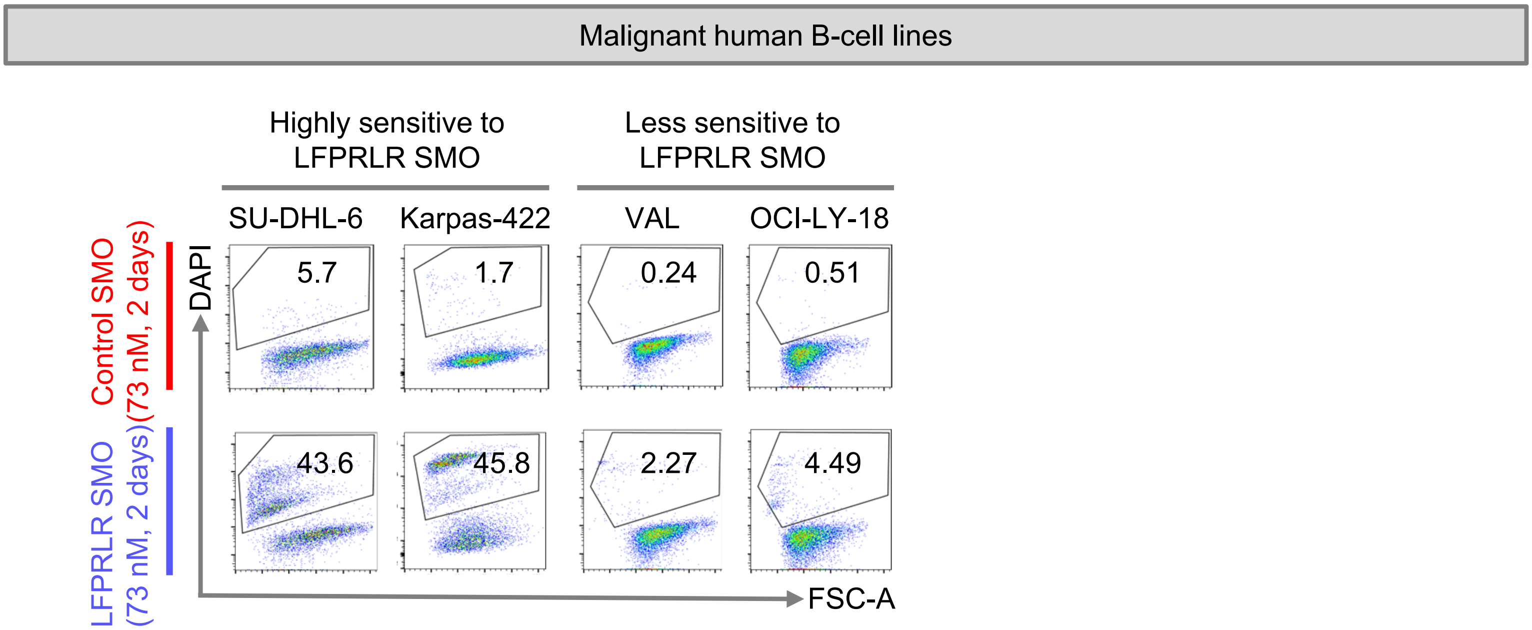

Supplementary Fig. 12: Knockdown of LFPRLR in malignant human B cells reduces their viability. Dead cells were assessed by measuring DAPI+ cells by flow cytometry in malignant human B-cell lines after treatment with 73nM control SMO or LFPRLR SMO for 48h.

Supplementary Fig. 13: *Expression of LF and IF PRLR in malignant human B cells*

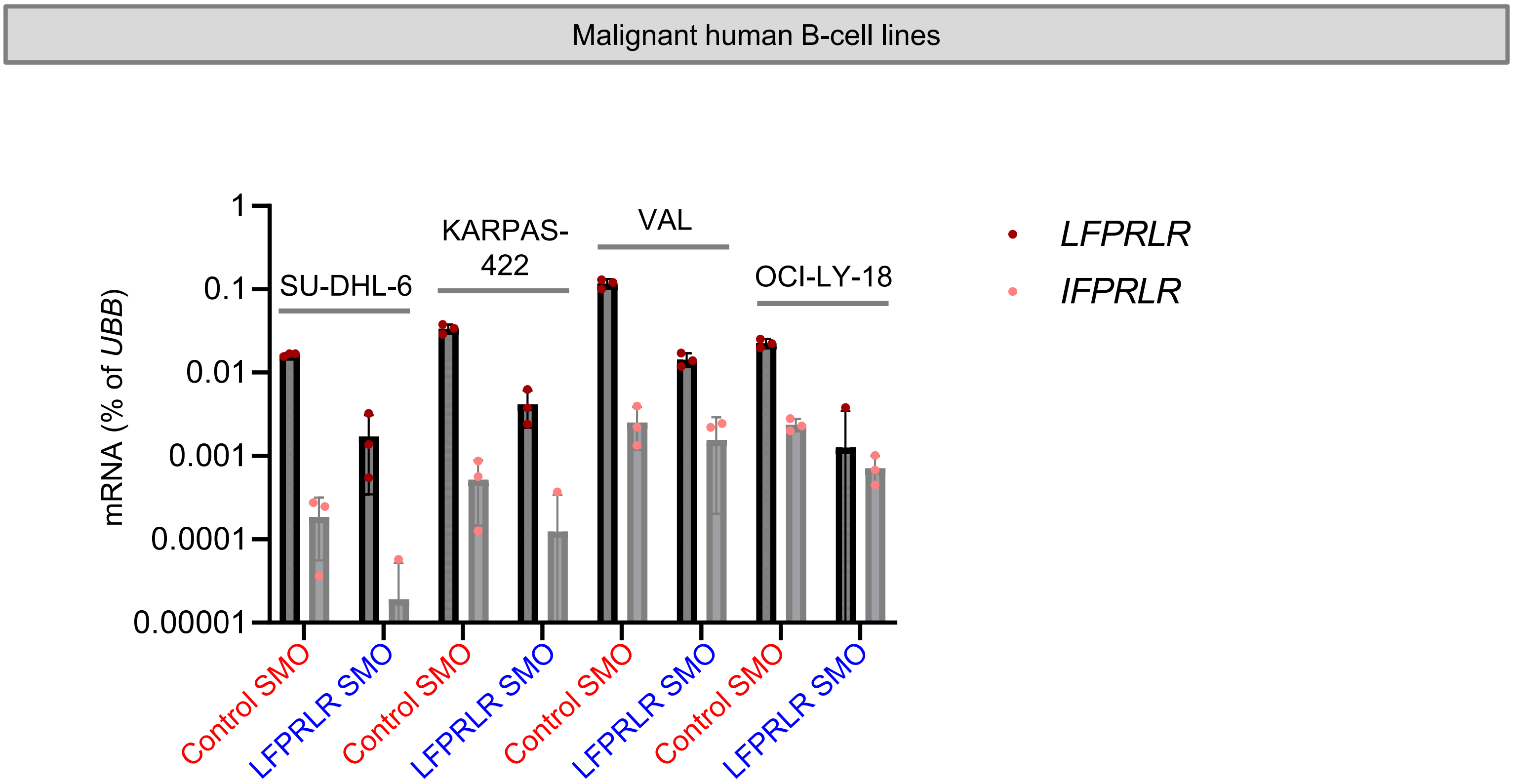

Supplementary Fig. 13: Expression of *LF* and *IFPRLR* in malignant human B cells. *LFPRLR* (red dots) and *IFPRLR* (pink dots) mRNA levels in malignant human B-cell lines (SU-DHL-6, Karpas-422, VAL and OCI-LY18) treated with their IC50 concentration of control SMO or LFPRLR SMO for 48h. Each qPCR sample was run in 3 technical replicates. For bars where <3 dots are shown, the remaining values are zero and could not be plotted on log scale. *UBB* was used as the house keeping gene in qPCR.

Supplementary Fig. 14: *Full scan of blot shown in Figure 5j*

a

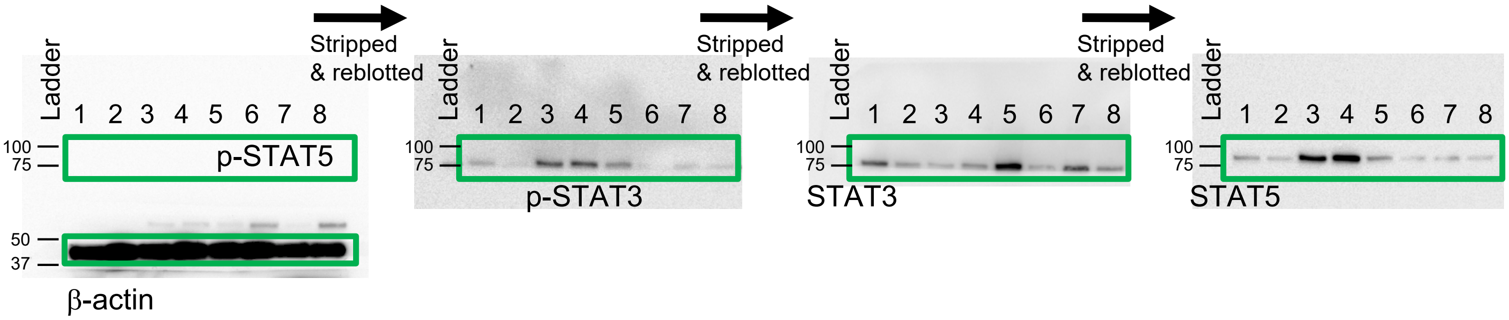

Lanes Left to right: Ladder

1. SU-DHL-6 + Control SMO (48h)
2. SU-DHL-6 + LFPRLR SMO (48h)
3. Karpas-422 + Control SMO (48h)
4. Karpas-422 + LFPRLR SMO (48h)
5. VAL + Control SMO (48h)
6. VAL + LFPRLR SMO (48h)
7. OCI-LY18 + Control SMO (48h)
8. OCI-LY18 + LFPRLR SMO (48h)

b

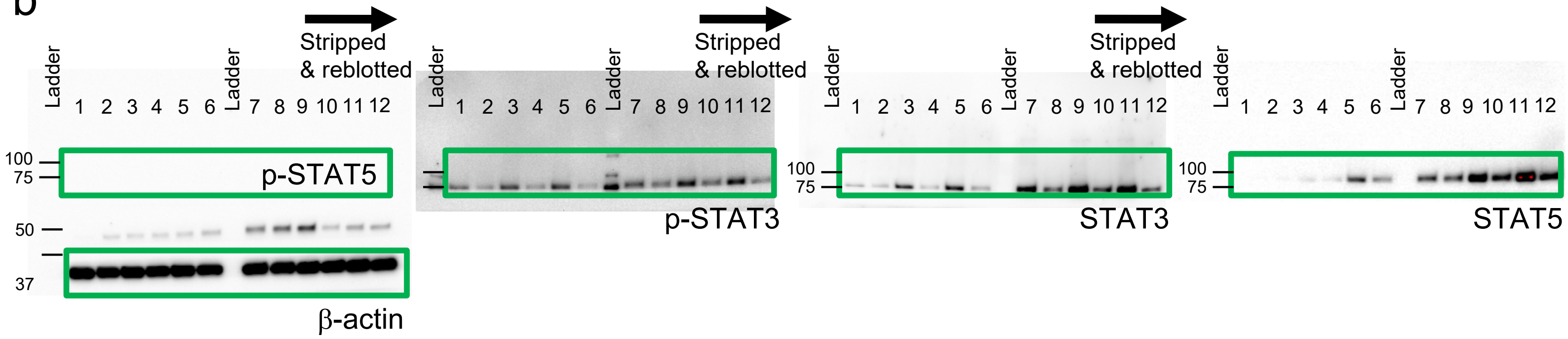

Lanes Left to right: Ladder

1. SU-DHL-6 + Control SMO (3h)
2. SU-DHL-6 + LFPRLR SMO (3h)
3. SU-DHL-6 + Control SMO (8h)
4. SU-DHL-6 + LFPRLR SMO (8h)
5. SU-DHL-6 + Control SMO (12h)
6. SU-DHL-6 + LFPRLR SMO (12h)

Ladder

7. OCI-LY18 + Control SMO (3h)
8. OCI-LY18 + LFPRLR SMO (3h)
9. OCI-LY18 + Control SMO (8h)
10. OCI-LY18 + LFPRLR SMO (8h)
11. OCI-LY18 + Control SMO (12h)
12. OCI-LY18 + LFPRLR SMO (12h)

c

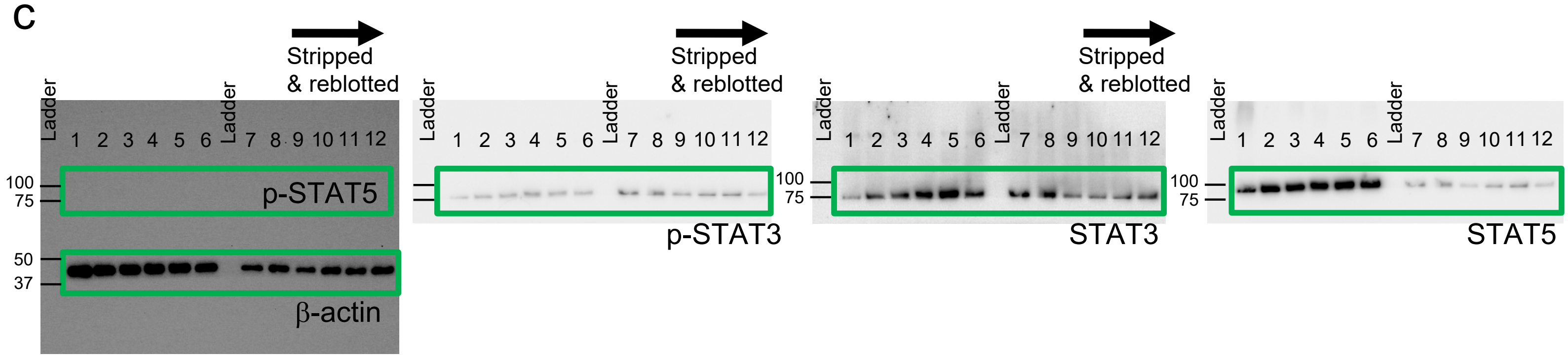

Lanes Left to right: Ladder

1. Karpas-422 + Control SMO (3h)
2. Karpas-422 + LFPRLR SMO (3h)
3. Karpas-422 + Control SMO (8h)
4. Karpas-422 + LFPRLR SMO (8h)
5. Karpas-422 + Control SMO (12h)
6. Karpas-422 + LFPRLR SMO (12h)

Ladder

7. VAL + Control SMO (3h)
8. VAL + LFPRLR SMO (3h)
9. VAL + Control SMO (8h)
10. VAL + LFPRLR SMO (8h)
11. VAL + Control SMO (12h)
12. VAL + LFPRLR SMO (12h)

Supplementary Fig. 14: Full scan of blot shown in Figure 5j. Blot was developed in ChemiDoc MP imaging system (BioRad). Membrane was re-blotted for similar molecular weight proteins using stripping buffer.

Supplementary Fig. 15: *Changes in STAT5 activation and level after LFPRLR knockdown in malignant B cells*

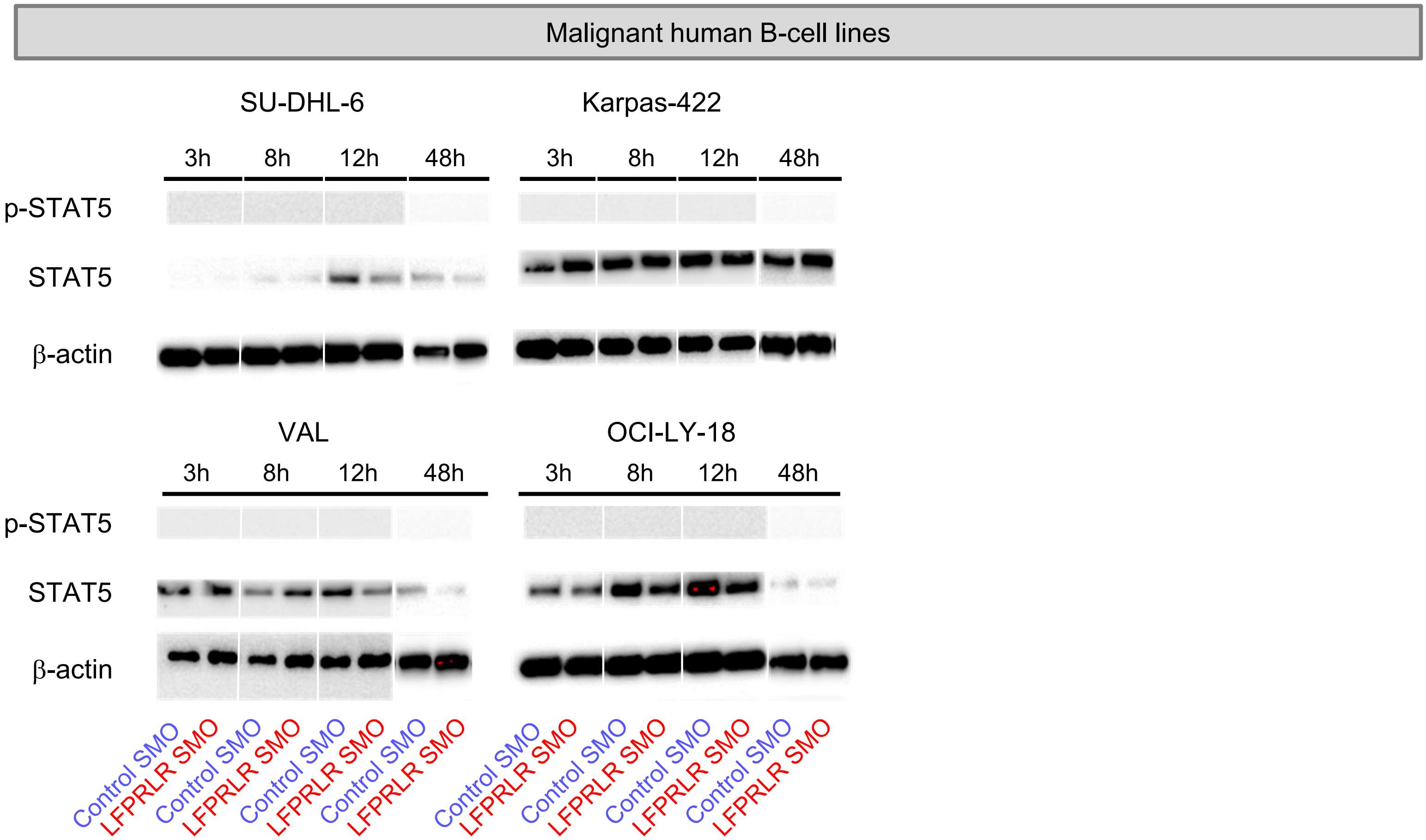

Supplementary Fig. 15: Changes in STAT5 activation and level after LFPRLR knockdown in malignant B cells. Immunoblotting showing reduction in global STAT5 levels in three out of four malignant B cell lines treated at their IC50 concentrations of LFPRLR SMO for 12 hours. β-actin was used as the loading control in immunoblotting.

Supplementary Fig. 16: *Neutralization of PRL secreted by malignant human B cells reduces their viability*

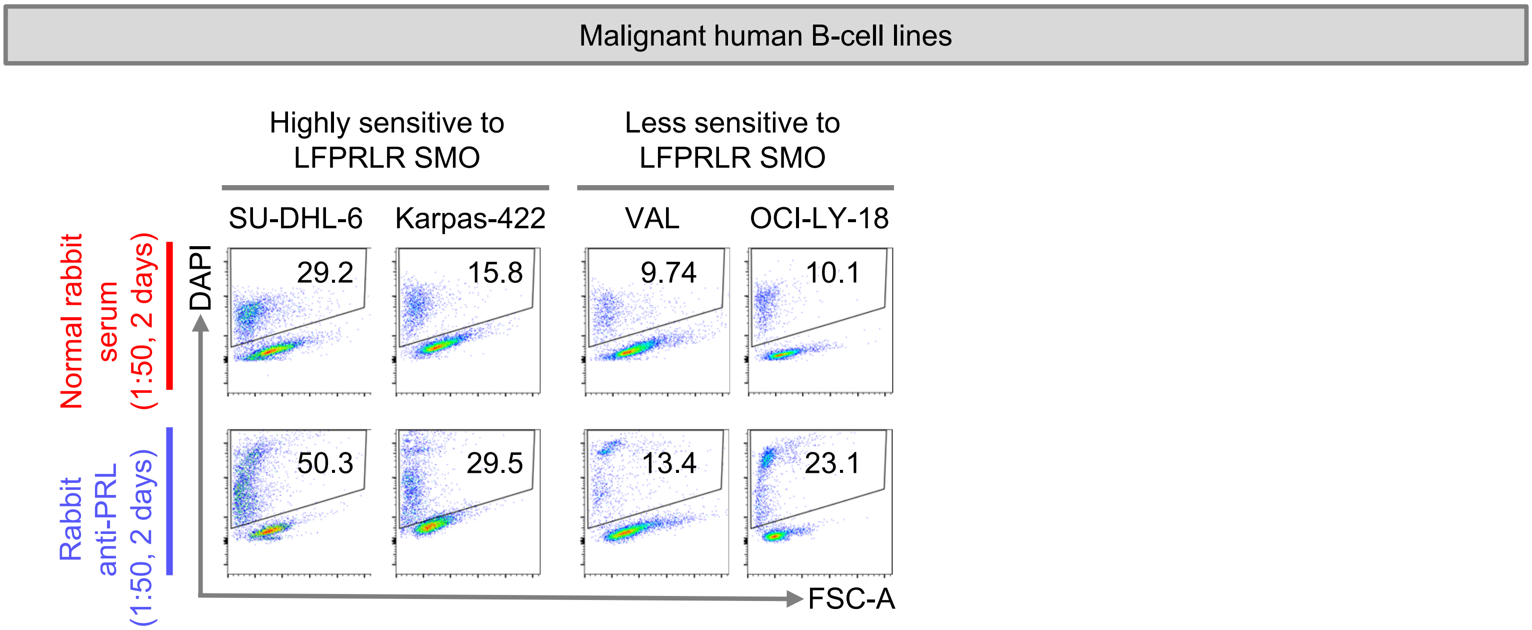

Supplementary Fig. 16: Neutralization of PRL secreted by malignant human B cells reduces their viability. DAPI+ dead cells were assessed by flow cytometry in malignant human B-cell lines after treatment with 1:50 dilution of normal rabbit serum or rabbit anti-human PRL for 48h.

Supplementary Fig. 17: *Full scan of blot shown in Figs. 7e- f*

Full scan of blot shown in Figure 7e

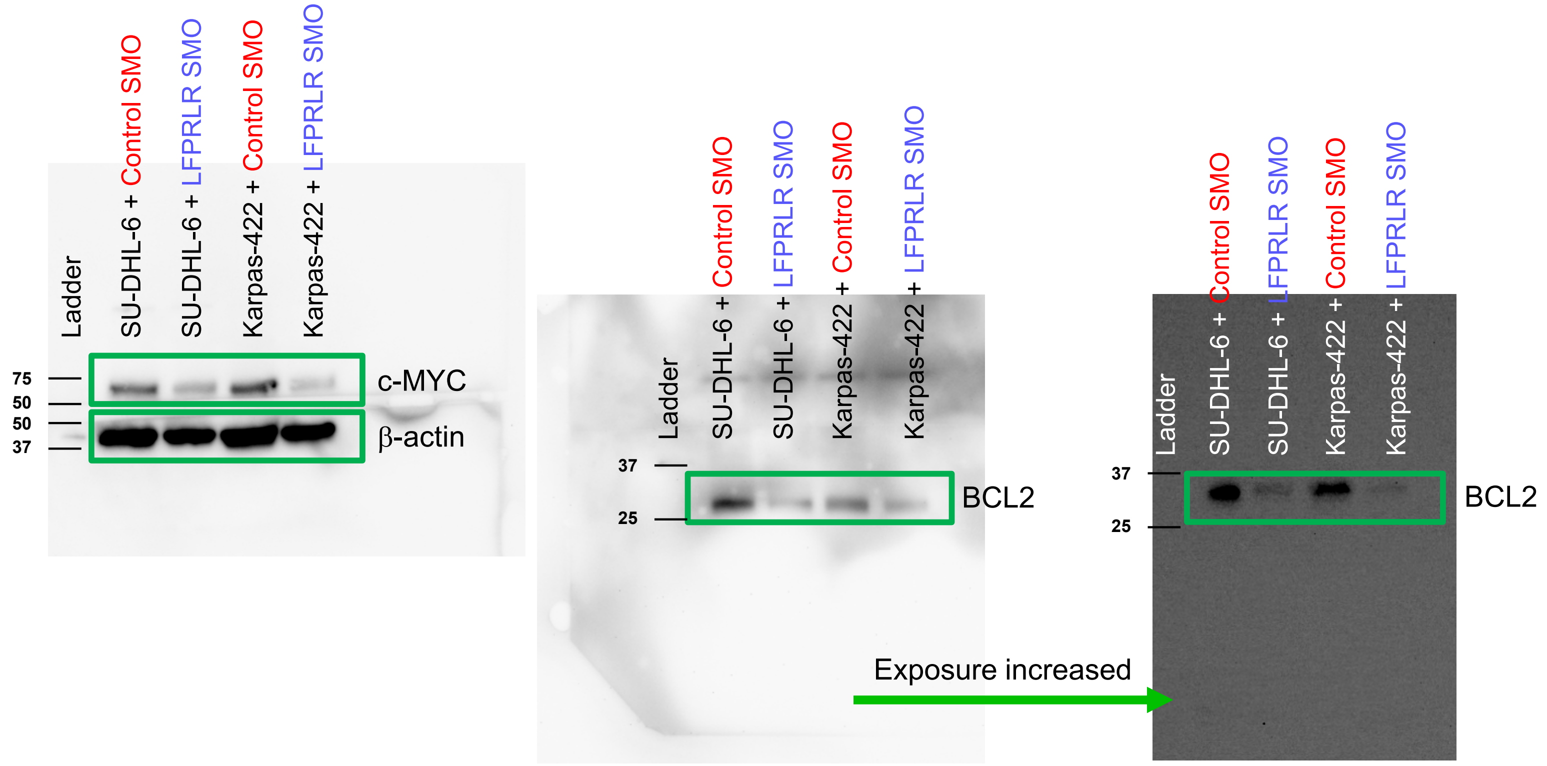

Full scan of blot shown in Figure 7f

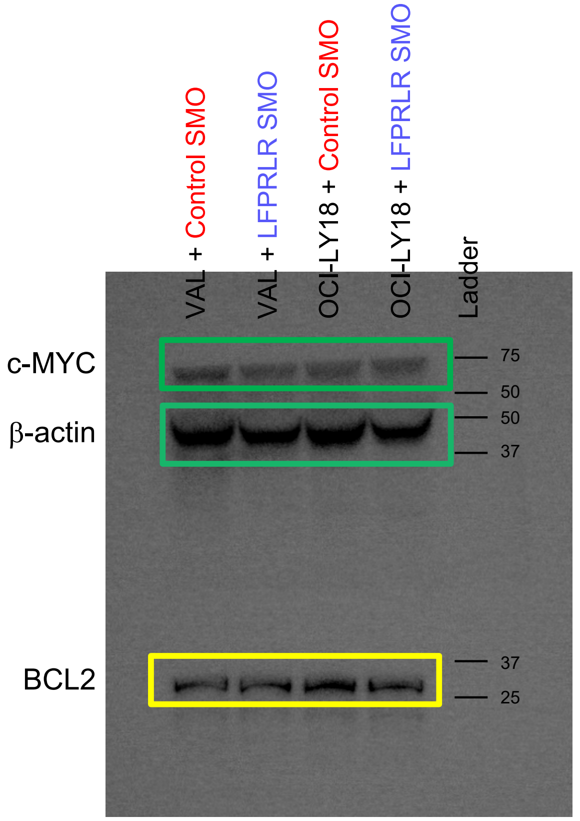

Supplementary Fig. 17: Full scan of blot shown in Figs. 7e-f. Blot was developed in ChemiDoc MP imaging system (BioRad)

Supplementary Table 1: *List of oligonucleotide sequences used in the study*

a. Splice modulating oligomer (SMO)

SMO	Sequence
Control SMO	5’-AGACGAGATTCGATCGGAGTA-3’
mLFPRLR SMO	5’-GCCCTTCTATTGAAACACAGATACA-3’
hLFPRLR SMO	5’-GCCCTTCTATTAAAACACAGACACA-3’

b. Quantitative RT-PCR (m= mouse, h= human)

Primer	Sequence
m*Ubb*- F	5’-AGCCCAGTGTTACCACCAAG-3’
m*Ubb*- R	5’-ACCCAAGAACAAGCACAAGG-3’
m*LF Prlr*- F	5’-ATAAAAGGATTTGATACTCATCTGCTAGAG-3’
m*LF Prlr*- R	5’-TGTCATCCACTTCCAAGAACTCC-3’
m*SF1 Prlr*- F	5’-AAGCCAGACCATGGATACTGGAG-3’
m*SF1 Prlr*- R	5’-AACTGGAGAATAGAACACCAGAG-3’
m*SF2 Prlr*- F	5’-TGCATCTTTCCACCAGTTCCGGGGC-3’
m*SF2 Prlr*- R	5’-TCAAGTTGCTCTTTGTTGTCAAC-3’
m*SF3 Prlr*- F	5’-TGCATCTTTCCACCAGTTCCGGGGC-3’
m*SF3 Prlr*- R	5’-TTGTATTTGCTTGGAGAGCCAGT-3’
m*Myc*- F	5’-TCGCTGCTGTCCTCCGAGTCC-3’
m*Myc*- R	5’-GGTTTGCCTCTTCTCCACAGAC-3’
m*Bcl2*- F	5’-CCTGTGGATGACTGAGTACCTG-3’
m*Bcl2*- R	5’-AGCCAGGAGAAATCAAACAGAGG-3’
m*Aicda*- F	5’-AAATGTCCGCTGGGCCAA-3’
m*Aicda*- R	5’-CATCGACTTCGTACAAGGG-3’
h*UBB*- F	5’-GCCGCACTCTTTCTGACTACAAC-3’
h*UBB*- R	5’-ACCTCCAGAGTGATGGTCTTGC-3’
h*LFPRLR*- F	5’-TCCAGGTATGTGGGTTTCAT-3’
h*LFPRLR*- R	5’-GATTTGATGCTCATCTGTTGGA-3’
h*IFPRLR*-F	5’-CTGTTCTCTCTCTTGACTTGGG-3’
h*IFPRLR*-R	5’-GATTTGATGCTCATCTGTTGGA-3’
h*SF1aPRLR*- F	5’-TGGACTGTGGTCAATGTTGC-3’
h*SF1aPRLR*- R	5’-GATAGTGAGGACCAGCATCTAATG-3’
h*SF1bPRLR*- F	5’-CAACATCAAGGGGTCACCTC-3’
h*SF1bPRLR*- R	5’-CATGAATGATACAACCGTGTGG-3’
h*MYC*- F	5’-CCTGGTGCTCCATGAGGAGAC-3’
h*MYC*- R	5’-CAGACTCTGACCTTTTGCCAGG-3’
h*PRL*- F	5’-GAGGAGCAAACCAAACGGCTTC-3’
h*PRL*- R	5’-AAGGCGAGACTCTTCATCAGCC-3’
h*BCL2*- F	5’-ATCGCCCTGTGGATGACTGAGT-3’
h*BCL2*- R	5’-GCCAGGAGAAATCAAACAGAGGC-3’

c. *mIGH* sequencing

Primer	Sequence
V_H1- F	5’-AAGGCCACACTGACTGTAGAC-3’
Cµ- R	5’-TGGCCACCAGATTCTTATCAG-3’

Supplementary Table 2: *List of antibodies used in the study*

a. Flow cytometry

Antibodies	Clone ID	Specificity	Dilution	Source
Anti-CD19	1D3/CD19	Mouse	7:1000	Biolegend
Anti-CD45R/B220	RA3-6B2	Mouse/Human	5:1000	Biolegend
Anti-CD138 (Syndecan-1)	281-2	Mouse	1:100	Biolegend
Anti-Blimp-1	5E7	Mouse	1:100	Biolegend
Anti-AID	mAID-2	Mouse/Human	1:100	eBioscience
Anti-BCL2	BCL/10C4	Mouse/Rat	1:100	Biolegend
Anti-CD3	UCHT1	Human	1:100	Biolegend
Anti-CD56	5.1H11	Human	1:100	Biolegend
Anti-CD19	HIB19	Human	1:100	Biolegend
Anti-CD4	RM4-5	Mouse	1:100	Biolegend
Anti-CD19	1D3	Mouse	1:100	BD
Anti-CD11c	N418	Mouse	1:100	Biolegend
Anti-CD8	5306.7	Mouse	1:100	Biolegend
Anti-CD3	17A2	Mouse	1:100	BD
Anti-PDCA1	927	Mouse	1:100	BD
Anti-NKP46	29A1.4	Mouse	1:100	BD
Ghost Dye™ UV 450			1:100	Tonbo
Anti-TCL1	1-21	Human	1:100	Biolegend

b. Immunoblotting

Antibodies	Clone ID	Specificity	Dilution	Source
Anti-cMYC	D84C12	Mouse/Human	1:1000	Cell Signaling Technology
Anti-β-Actin	8H10D10	Mouse/Human	1:500	Cell Signaling Technology
Anti-BCL2	D17C4	Mouse/Human	1:1000	Cell Signaling Technology
Anti-STAT5	D2O6Y	Mouse/Human/Rat	1:1000	Cell Signaling Technology
Anti-STAT3	124H6	Mouse/Human/Rat	1:1000	Cell Signaling Technology
Anti-phospho STAT3 (Tyr705)		Mouse/Human/Rat	1:1000	Cell Signaling Technology
Anti-phospho STAT5 (Tyr694)	14H2	Mouse/Human	1:1000	Cell Signaling Technology
