# Supplementary material for: Isoform-specific knockdown of long and intermediate prolactin receptors interferes with evolution of B-cell neoplasms
Source: Commun Biol. 2023 Mar 20;6:295. doi: 10.1038/s42003-023-04667-8 (PMC10027679; doi:10.1038/s42003-023-04667-8)
Supplement: Supplementary file 3 — Description of Additional Supplementary Files [file 42003_2023_4667_MOESM3_ESM.pdf]

Description of Additional Supplementary Files

File name: Supplementary Data 1

Description: Numerical source data behind Main Figs. 1-7 in the paper.
