# Supplementary material for: Isoform-specific knockdown of long and intermediate prolactin receptors interferes with evolution of B-cell neoplasms
Source: Commun Biol. 2023 Mar 20;6:295. doi: 10.1038/s42003-023-04667-8 (PMC10027679; doi:10.1038/s42003-023-04667-8)
Supplement: Supplementary file 4 — Reporting Summary-Taghi Khani et al [file 42003_2023_4667_MOESM4_ESM.pdf]

nature portfolio

Corresponding author(s): Srividya Swaminathan
Ameae M. Walker

Last updated by author(s): Feb 16, 2023

Reporting Summary

Nature Portfolio wishes to improve the reproducibility of the work that we publish. This form provides structure for consistency and transparency in reporting. For further information on Nature Portfolio policies, see our Editorial Policies and the Editorial Policy Checklist.

Statistics

For all statistical analyses, confirm that the following items are present in the figure legend, table legend, main text, or Methods section.

n/a	Confirmed	
☐	☒	The exact sample size (*n*) for each experimental group/condition, given as a discrete number and unit of measurement
☐	☒	A statement on whether measurements were taken from distinct samples or whether the same sample was measured repeatedly
☐	☒	The statistical test(s) used AND whether they are one- or two-sided<br>*Only common tests should be described solely by name; describe more complex techniques in the Methods section.*
☐	☒	A description of all covariates tested
☐	☒	A description of any assumptions or corrections, such as tests of normality and adjustment for multiple comparisons
☐	☒	A full description of the statistical parameters including central tendency (e.g. means) or other basic estimates (e.g. regression coefficient) AND variation (e.g. standard deviation) or associated estimates of uncertainty (e.g. confidence intervals)
☐	☒	For null hypothesis testing, the test statistic (e.g. *F*, *t*, *r*) with confidence intervals, effect sizes, degrees of freedom and *P* value noted<br>*Give P values as exact values whenever suitable.*
☒	☐	For Bayesian analysis, information on the choice of priors and Markov chain Monte Carlo settings
☒	☐	For hierarchical and complex designs, identification of the appropriate level for tests and full reporting of outcomes
☒	☐	Estimates of effect sizes (e.g. Cohen's *d*, Pearson's *r*), indicating how they were calculated

Our web collection on statistics for biologists contains articles on many of the points above.

Software and code

Policy information about availability of computer code

Data collection	List of the softwares we used:<br>Flow cytometry acquisition:BD FACSymphony, BD FACSDiva™ Software<br>Quantitative Real Time PCR: QuantStudio 7 flex real-time PCR system (Applied Biosystems), QuantStudio™ Real-Time PCR Software v1.7.2<br>Next generation sequencing (NGS): MiSeq Illumina 2x150 bp platform
Data analysis	List of the softwares we used:<br>Flow cytometry: FlowJo10.7.1<br>RNA sequencing: STAR v.2.7.6a, R package DESeq2<br>Next generation sequencing (NGS): IMGTHighV-QUEST. VDJTools (v1.1.7)<br>Plots: GraphPad Prism 9

For manuscripts utilizing custom algorithms or software that are central to the research but not yet described in published literature, software must be made available to editors and reviewers. We strongly encourage code deposition in a community repository (e.g. GitHub). See the Nature Portfolio guidelines for submitting code & software for further information.

Data

Policy information about availability of data

All manuscripts must include a data availability statement. This statement should provide the following information, where applicable:

- Accession codes, unique identifiers, or web links for publicly available datasets
- A description of any restrictions on data availability
- For clinical datasets or third party data, please ensure that the statement adheres to our policy

Next generation sequencing of the immunoglobulin heavy chain repertoire described in this study has been deposited in Gene Expression Omnibus under accession GSE207186. This data set will be made public on May 1, 2023.
We employed previously published data sets available in GEO to conduct survival analysis as described in Fig. 5a and Supplementary Fig. 9. The following data sets were used: GSE4475, GSE10846, and E-TABM-346.
We also used RNA-sequencing data of EGAS00001003266, St. Jude, for measurement of PRL (Fig. 5b) and LF+IFPRLR: total PRLR mRNA (Supplementary Fig. 10).

Human research participants

Policy information about studies involving human research participants and Sex and Gender in Research.

Reporting on sex and gender	NA
Population characteristics	NA
Recruitment	NA
Ethics oversight	NA

Note that full information on the approval of the study protocol must also be provided in the manuscript.

Field-specific reporting

Please select the one below that is the best fit for your research. If you are not sure, read the appropriate sections before making your selection.

☒ Life sciences ☐ Behavioural & social sciences ☐ Ecological, evolutionary & environmental sciences

For a reference copy of the document with all sections, see nature.com/documents/nr-reporting-summary-flat.pdf

Life sciences study design

All studies must disclose on these points even when the disclosure is negative.

Sample size	For experiments in primary MRL-lpr and TCL1-tg mice, to ensure reproducibility and rigor, give sufficient room for unexpected loss of mice, and account for variation between individual mice within a group, we employed 28 female MRL-lpr mice (14 in each group of treatment) and male and female TCL1tg mice (8 males and 8 females in each group). Because SLE occurs more frequently in females, we used only female MRL-lpr SLE-prone mice for our studies. This justification is provided in the manuscript.<br>For in vivo cell-line derived xenograft studies, 6 female, 7-week-old, NSG mice were used per group. Minimum mouse number calculation was done based on our previously published studies to ensure reproducibility and rigor.<br>For in vitro experiments, we did not perform sample size calculation.
Data exclusions	No data exclusions in the study.
Replication	For in vivo experiments involving primary mouse models of SLE and DLBCL, reproducibility of the results was ensured by drawing mice from at least three independent breedings for each experiment. NSG transplant recipient mice used for in vivo cell-line derived xenograft studies were identical (7-weeks of age) in all groups at the time of CDX transplantation.<br>All in vitro experiments were conducted with at least three different biological replicates, drawn from independent batches/passages of cells. For qPCR studies, each biological replicate was run as three technical replicates to account for handling errors.
Randomization	Mice were randomly assigned to control SMO or LFPRLR SMO groups in both MRL-lpr and TCL1-tg mice models.
Blinding	No blinding was possible in experiments involving mice if the same person treated mice, processed the samples, and analyzed them.

Reporting for specific materials, systems and methods

We require information from authors about some types of materials, experimental systems and methods used in many studies. Here, indicate whether each material, system or method listed is relevant to your study. If you are not sure if a list item applies to your research, read the appropriate section before selecting a response.

Materials & experimental systems

n/a	Involved in the study
☐	☒ Antibodies
☐	☒ Eukaryotic cell lines
☒	☐ Palaeontology and archaeology
☐	☒ Animals and other organisms
☒	☐ Clinical data
☒	☐ Dual use research of concern

Methods

n/a	Involved in the study
☒	☐ ChIP-seq
☐	☒ Flow cytometry
☒	☐ MRI-based neuroimaging

Antibodies

Antibodies used

Refer to Supplementary Information: Supplementary Table 2
a. List of antibodies used in the Flow Cytometry
Antibodies, Dilution, Source
Anti-CD19, 7:1000, Biolegend
Anti-CD45R/B220, 5:1000, Biolegend
Anti-CD138 (Syndecan-1), 1:100, Biolegend
Anti-Blimp-1, 1:100, Biolegend
Anti-AID, 1:100, eBioscience
Anti-BCL2, 1:100, Biolegend
Anti-CD3, 1:100, Biolegend
Anti-CD56, 1:100, Biolegend
Anti-CD19, 1:100, Biolegend
Anti-CD4, 1:100, Biolegend
Anti-CD19, 1:100, BD
Anti-CD11c, 1:100, Biolegend
Anti-CD8, 1:100, Biolegend
Anti-CD3, 1:100, BD
Anti-PDCA1, 1:100, BD
Anti-NKP46, 1:100, BD
Ghost Dye™ UV 450, 1:100, Tonbo
Anti-TCL1, 1:100, Biolegend

b. List of antibodies used in the immunoblotting
Antibodies, Dilution, Source
Anti-cMYC, 1:1000, Cell Signaling Technology
Anti-β-Actin, 1:500, Cell Signaling Technology
Anti-BCL2, 1:1000, Cell Signaling Technology
Anti-STAT3, 1:1000, Cell Signaling Technology
Anti-STAT5, 1:1000, Cell Signaling Technology
Anti-phospho STAT3 (Tyr705), 1:1000, Cell Signaling Technology
Anti-phospho STAT5 (Tyr694), 1:1000, Cell Signaling Technology

c. PRL neutralizing antibody experiment: normal rabbit serum or rabbit anti-PRL (NIDDK standard, AFP55762089)
d. PRL antibody for detection by ELISA: Biotechne Quantikine ELISA kit Cat #DPRL00

Validation

All antibodies in the study have been validated by the manufacturer and have been used according to manufacturer's instructions. Refer to Methods sections: 'Flow cytometry' and 'Immunoblotting'.

Eukaryotic cell lines

Policy information about cell lines and Sex and Gender in Research

Cell line source(s)

Methods: Refer to section 'Cell lines and cell culture'. Mycoplasma-negative human cell lines were obtained from DSMZ (Deutsche Sammlung von Mikroorganismen und Zellkulturen)

Authentication

Human cell lines were obtained from DSMZ (https://www.dsmz.de/) that provides authentication documents for each line. Therefore, none of these cell lines used were authenticated for this study.

Mycoplasma contamination

Yes, only cell lines negative for mycoplasma were used. See Methods: Cell lines and culture

Commonly misidentified lines
(See ICLAC register)

No misidentified lines were used in the study.

Animals and other research organisms

Policy information about studies involving animals; ARRIVE guidelines recommended for reporting animal research, and Sex and Gender in Research

Laboratory animals	Refer to Methods section"Animal models", Animal studies were conducted in compliance with Institutional Animal Care and Use Committees at City of Hope and University of California, Riverside. 6-week-old female SLE-prone MRL-lpr mice homozygous for the Fas cell surface death receptor mutation (Faslpr) were anesthetized with isoflurane, and Alzet minipumps (Durect, Cupertino, CA) were implanted subcutaneously between the scapulae. Mice were randomly assigned to control SMO or LFPRLR SMO groups and coded by ear punch. Animals in each group were housed individually after pump implantation until wound clips were removed. Alzet pumps that delivered 100 pmoles/h/mouse of either control SMO or LFPRLR SMO were changed after 4 weeks. At week 8 of treatment, two hours before euthanasia, each animal received an intraperitoneal injection of 2.8 mg of the nucleoside analog, 5-ethynyl-2′-deoxyuridine (EdU). 8-week-old male and female DLBCL-prone TCL1-tg mice were implanted with Alzet minipumps and treated with control or LFPRLR SMO for 8 weeks, as described for MRL-lpr mice.<br>Immune-deficient NSG mice were used as transplant recipients in the human B-cell malignancy CDX models. Treatment with SMOs in the NSG CDX-recipient mice was conducted as described above for the primary mouse models of SLE and DLBCL, except that the treatment duration was shorter to ensure tumor size remained within guidelines in the control SMO treated animals. Transplant recipient CDX mice were euthanized following the stringent guidelines on tumor volume and health of mice laid down by IACUC of City of Hope.
Wild animals	NA
Reporting on sex	We only used female MRL-lpr mice because SLE is more frequent in females. We used female NSG mice for CDX model experiments. In experiments involving pre-malignant or malignant B cells, we represented both sexes equally because B-cell malignancies develop at the same frequencies in both males and females.
Field-collected samples	NA
Ethics oversight	Animal studies were conducted in compliance with Institutional Animal Care and Use Committees at City of Hope and University of California, Riverside.

Note that full information on the approval of the study protocol must also be provided in the manuscript.

Flow Cytometry

Plots

Confirm that:

☒ The axis labels state the marker and fluorochrome used (e.g. CD4-FITC).

☒ The axis scales are clearly visible. Include numbers along axes only for bottom left plot of group (a 'group' is an analysis of identical markers).

☒ All plots are contour plots with outliers or pseudocolor plots.

☒ A numerical value for number of cells or percentage (with statistics) is provided.

Methodology

Sample preparation	Cells were thawed in Complete RPMI and stained with fluorochrome-tagged surface antibodies and Ghost-UV450 for 30 min on ice. Using eBioscience™ Transcription Factor Staining Buffer Set, cells were fixed, permeabilized, and stained with intracellular antibodies for 30 min on ice followed by acquisition on the BD FACSymphony cytometer. Gates were set using single-stained and fluorescence minus one (FMO) controls.
Instrument	BD FACSymphony
Software	FlowJo10.7.1
Cell population abundance	We verified B cells sorted out magnetically using CD19 microbeads by Flow Cytometry (Supplementary Fig. 5).<br>We showed verification of post-sort purity of magnetically sorted B, T and NK cells from PBMC of healthy donors by flow cytometry (Supplementary Fig. 11).
Gating strategy	Gating strategy for flow cytometry analysis of mouse splenic WBCs (Supplementary Fig. 1):<br>From the lymphocyte cluster; singlets were gated followed by selection of live (Ghost-UV450-) populations. B cells, T cells, and non-B non-T cells were then gated on CD19+, CD3+, and CD19- CD3-, respectively. T cells were gated on CD8+, CD4+, and CD4-CD8- T cells. pDCs and cDCs were gated on CD11c and PDCA1 after gating out NKp46+ NK cells from the non-B non-T fraction.<br><br>Gating strategy for flow cytometry by intracellular staining (Supplementary Fig. 3):<br>From the lymphocyte cluster; singlets were gated followed by selection of live (Ghost-UV450-) populations. (a)Total CD138+ plasma cells, CD138+B220+ plasmablasts, and CD138+B220- LLPC were then measured by gating on Blimp1+cells. (b-d) Total

CD19+ B cells were then gated and BCL2 (b), or AID (c), or TCL1 (d) was measured within these cells. Gates for all markers were set based on fluorescence minus one controls (FMO).

Gating strategy for flow cytometry analysis of human peripheral blood B, T and NK cells (Supplementary Fig. 11a):
From the lymphocyte cluster; singlets were gated followed by selection of live (Ghost-UV450-) populations. B cells were then gated on CD3- CD19+, T cells were gated on CD3+ CD19- and NK cells were then gated on CD3-CD56+

☒ Tick this box to confirm that a figure exemplifying the gating strategy is provided in the Supplementary Information.
